# Exploring negative symptoms heterogeneity in patients diagnosed with schizophrenia and schizoaffective disorder using cluster analysis

**DOI:** 10.1186/s12888-023-05101-3

**Published:** 2023-08-15

**Authors:** Feten Fekih-Romdhane, Romy Hajje, Chadia Haddad, Souheil Hallit, Jocelyne Azar

**Affiliations:** 1 The Tunisian Center of Early Intervention in Psychosis, Department of Psychiatry “Ibn Omrane”, Razi hospital, Manouba, 2010 Tunisia; 2 Faculty of Medicine of Tunis, Tunis El Manar University, Tunis, Tunisia; 3 Faculty of Science, Lebanese University, Fanar, Lebanon; 4 Research Department, Psychiatric Hospital of the Cross, Jal Eddib, Lebanon; 5 INSPECT-LB (Institut National de Santé Publique, d’Épidémiologie Clinique et de Toxicologie-Liban), Beirut, Lebanon; 6 School of Health Sciences, Modern University for Business and Science, Beirut, Lebanon; 7 School of Medicine and Medical Sciences, Holy Spirit University of Kaslik, P.O. Box 446, Jounieh, Lebanon; 8 Applied Science Research Center, Applied Science Private University, Amman, Jordan; 9 School of Medicine, Lebanese American University, Byblos, Lebanon

**Keywords:** Psychosis, Schizophrenia, Heterogeneity, Negative symptoms, Cluster analysis

## Abstract

**Background:**

Dissecting the heterogeneity of schizophrenia may help foster progress in understanding its etiology and lay the groundwork for the development of new treatment options for primary or enduring negative symptoms (NS). In this regard, the present study aimed to: (1) to use cluster analysis to identify subgroups of Lebanese patients diagnosed with either schizophrenia or schizoaffective disorder based on NS clusters, and (2) to relate the statistically-derived subgroups to clinically relevant external validators (including measures if state and trait depression, stigma, insight, loneliness, social support).

**Method:**

A total of 202 adult long-stay, chronic, and clinically remitted patients (166 diagnosed with schizophrenia and 36 with schizoaffective disorder) were enrolled. A cluster analysis approach was adopted to classify patients based on the five NS domains social withdrawal, emotional withdrawal, alogia, avolition and anhedonia.

**Results:**

A three-cluster solution was obtained based on unique NS profiles, and divided patients into (1) low NS (LNS; 42.6%) which characterized by the lowest mean scores in all NS domains, (2) moderate NS (MNS; 25.7%), and (3) high NS (HNS; 31.7%). Post-hoc comparisons showed that depression (state and trait), loneliness and social support could accurately distinguish the schizophrenia subgroups. Additionally, individuals in the HNS cluster had longer duration of illness, longer duration of hospitalization, and were given higher dosages of antipsychotic medication compared to those in the other clusters, but these differences did not achieve the statistical significance.

**Conclusion:**

Findings provide additional support to the categorical model of schizophrenia by confirming the existence of three alternate subtypes based on NS. The determination of distinct NS subgroups within the broad heterogeneous population of people diagnosed with schizophrenia may imply that each subgroup possibly has unique underlying mechanisms and necessitates different treatment approaches.

## Introduction

According to the latest estimates of the World Health Organization, schizophrenia affects approximately 1 in 300 people or 24 million people worldwide [1], with the annual incidence being steadily on the rise in certain regions [2, 3]. Although it is not as common as many other mental disorders, and despite recent advances in psychiatry, it remains one of the most debilitating disorders. Indeed, schizophrenia is associated with high rates of suicidality [4], excess early mortality [5], and impaired quality of life [6]. In addition, existing evidence indicates that only nearly one in five patients with schizophrenia report achieving clinical recovery [7], and one in seven will achieve functional recovery [8]. This variability in outcomes was partly attributable to substantial and hardly to delineate clinical heterogeneity of schizophrenia [9]. Several researchers proposed that schizophrenia represents a heterogeneous disorder with a group of distinct symptom structures and overlapping clinical phenotypes, rather than a unitary disease entity [10–12].

### Clinical heterogeneity of schizophrenia

The clinical heterogeneity of schizophrenia manifests in different symptom patterns, course trajectories, and treatment responses [13, 14]. Interestingly, these assumptions date back to Kraepelin’ s view of dementia praecox as “the expression of a single morbid process, though outwardly they often diverge very far from one another” [15], and Bleuler’ s labelling as “Group of Schizophrenias” [16]. Since the earliest conceptualizations of schizophrenia, there have been multiple efforts to develop subtypes that may help advance our understanding of the disease and its underlying pathogenesis [17–21]. The identification of schizophrenia subgroups also has many benefits in terms of informing “how to treat” based on a set of treatment choices (in contrast with the dimensional approach that rather informs the decision “to treat” or “not to treat”), and predicting response to treatment above and beyond symptoms severity and extent of suffering or disability [22]. However, traditional attempts to capture and map clinical heterogeneity of schizophrenia were unsuccessful and hampered by the lack of temporally stable and clinically valid taxonomic schemes. The subtyping scheme previously proposed in the Diagnostic and Statistical Manual of Mental Disorders (DSM) was dropped by the fifth edition because of its limited clinical validity, prognostic value, and research utility [23–25]. Researchers have, for example, individualized a putative “deficit” subtype with primary and persistent negative symptoms (NS) characterized by distinctive pathophysiology, clinical course, and treatment response [18, 26]. Although deficit schizophrenia seems to represent “the most viable candidate for a separable disease entity” within the disease [27], the categorical approach of NS is not uniformly accepted and some researchers lean more toward a dimensional approach where patients differ in the “amount” or “degree” rather than “status” [28].

In the DSM-5, NS are defined as one of the core dimensions of schizophrenia [29]. NS are common, with at least one NS being reported by over 50% of people with schizophrenia [30, 31]. They reflect an absence or reduction of functions that are normally present in the general population, and that are either related to expressive functions (e.g., alogia, blunted affect) or motivation and interest (e.g., asociality, anhedonia, avolition). This group of symptoms have greater impact on functioning than positive symptoms [32], and poses a significant burden on patients, their families, and the healthcare system. However, NS remain neglected targets for treatment [33], with most attention having rather been directed at treating positive symptoms that are more easily detectable and manageable [34]. Overall, the lack of therapeutic advances and mixed research findings about the underlying mechanisms of the negative symptomatic dimension of schizophrenia have emphasized the need for their reconceptualization [35]. Over the last years, considerable progress has been made in this direction. Studies have begun to acknowledge and provide valuable insight into the heterogeneity of NS (e.g., differentiating persistent vs. transient and primary vs. secondary NS) [36–38].

### Previous cluster analyses of NS

To better characterize the variable phenotypic expression of symptoms, Goldstein [39, 40] called for a clinically-informed and statistically supported nosology by using multivariate analysis (i.e. Cluster Analysis [41]) that enables identifying homogenous subgroups of patients within the broader schizophrenia diagnosis. To answer this call, numerous studies have applied this data-driven statistical approach to dissect the heterogeneity of schizophrenia. A recent systematic review of cluster and group-based studies showed that most of the existing studies addressed cognitive deficits, and could only find four cross-sectional studies assessing NS clusters [42]. These four studies depicted two to five patient trajectory groups, and identified a number of sociodemographic (e.g., gender, age, educational status, marital status) and clinical (e.g., duration of untreated psychosis, social functioning, quality of life, cognitive performance, depressive symptoms) predictors [42]. Among the four studies, two investigated NS in combination with positive symptoms and found three to four clusters (i.e., low scores on both positive and negative symptoms, high scores on both sets of symptoms, predominantly negative or positive symptoms scores) [43, 44]. In a different approach and method by Strauss et al. [45], NS were regarded as two separate factors instead of unitary NS scores and subsequently demonstrated two distinct symptom clusters, one characterized by predominant diminished expression while the other by higher anhedonia and avolition symptoms. The latter subgroup was marked by a poorer clinical and functional status [45].

For a better generalizability and a more optimal conceptualization of NS in schizophrenia, some studies have recently provided support for a latent five-factor model (i.e., blunted affect, alogia, anhedonia, asociality, and avolition) [46–49] that should be favored over both the one- and two-factor models [50]. This five-factor model of NS has initially been proposed in a consensus development conference held by the National Institute of Mental Health [51], and later its construct validity was consistently proven robust across different languages and cultures (e.g [47–49].,). Surprisingly however, the five NIMH consensus NS domains have only very recently been entered into a cluster analysis in a first and only study by Paul et al. [52]. Authors used a semi-structured interview designed to assess the five consensus domains (i.e., the Brief Negative Symptom Scale [53]), and could identify four distinct clusters within a sample of people diagnosed with schizophrenia or schizoaffective disorder: (1) low NS, (2), severe NS, (3) moderate NS with predominantly elevated blunted affect, and (4) moderate NS dominated by avolition [52]. The four clusters differed in their relationships with neurocognition, clinical characteristics, and functional outcome, thus suggesting that the NS subgroups established have distinct clinical and neuropsychological profiles [52].

### Rationale of the present study

In the present study, we sought to build on and extend prior research by providing some new insight into potential and unique NS subgroups within schizophrenia. We contribute to the existing literature in two ways. First, we respond to recent calls for research entering the latest five NIMH consensus NS domains into a cluster analysis in schizophrenia. We further add to the literature by using for the first time a self-report measure, i.e. the Self-Evaluation of Negative Symptoms (SNS) [54], in contrast with the previously used semi-structured interview (i.e. Brief Negative Symptom Scale (BNSS) [53]). There is some evidence on discrepancies between examiner-ratings and self-ratings for NS, with a majority of patients found to be unable to accurately report their symptoms in hetero-evaluation [55, 56]. In this regard, self-evaluation was proven to detect some clinical information on NS recognized and analyzed by the patients themselves that are not necessarily captured by clinicians in a standard interview [57]. Additionally, the SNS has proven to enable patients to express their speech expression, loss of emotion, and deficits in motivation regardless of their depressive symptoms [54, 58, 59]. Second, we investigate for the first time in this area a clinical population from an Arab country of the Middle East and North African (MENA) region. A recent systematic review revealed that the vast majority of studies available on clusters of schizophrenia symptoms were conducted in the USA and/or other Western countries [42]. Even though schizophrenia is a universal disease, affecting people all around the world regardless of origin, race, or culture social class [60], recent research suggests that it manifests differently across countries. Indeed, several studies pointed to marked variations in the expression and clinical trajectory of schizophrenia symptoms (including anhedonia, depressive symptoms, and emotional processing) depending on sociocultural, ethnic and contextual factors [61–64]. Hence, it is pertinent to provide the first data on the topic from a non-Western developing country of the largely under-researched MENA region, Lebanon. Therefore, our main objectives were: (1) to use cluster analysis to identify subgroups of Lebanese patients diagnosed with either schizophrenia or schizoaffective disorder based on NS clusters, and (2) to relate the statistically-derived subgroups to clinically relevant external validators (including measures if state and trait depression, stigma, insight, loneliness, social support). We hypothesized that clustering would allow us to accurately identify subgroups of patients characterized by severe and low levels of NS, and another intermediate subgroup with moderate levels of NS.

## Methods

### Participants

This study is a cross-sectional study. Our target population were patients with schizophrenia who were in a long-stay accommodation in the Psychiatric Hospital of the Cross, Jal Eddib (suburbs of the capital Beirut), Lebanon (The study population is described elsewhere [65, 66]). Inclusion criteria included the following: (1) being aged over 18 years, (2) diagnosed with schizophrenia or schizoaffective disorder by two independent psychiatrists according to the Diagnostic and Statistical Manual of Mental Disorders (DSM-5) criteria [35], (3) being a chronic patient, defined as with more than 1 year of illness duration [67], and institutionalized in the above-mentioned long-stay hospital for more than one year, (4) being considered clinically stable, as defined by Fleischhacker et al. [68]: patients “were required to be symptomatically stable, as judged by the treating physician, be receiving a stable dose of an antipsychotic drug for at least 4 weeks before the survey and be in good general physical health”, and (5) be able to recognize the objectives of the study and give their free consent to participate (in case of inability to consent a family member did) [69].

A total of 207 adult patients was identified at the hospital, 5 of which were excluded because of non-cooperation. The population consisted of 126 males and 76 females; 166 patients suffering from chronic schizophrenia and 36 from schizoaffective disorder.

#### Minimum sample size calculation

We used G*Power software to determine the minimum sample size; the latter was 137 participants, considering an alpha error of 5%, a power of 80%, a minimum R^2^ of 10% and allowing 7 predictors to be included in the final models.

### Data collection procedures and instruments

Data was obtained during an interview with patients at the hospital. The average time for the interview was around 30–45 min. The questionnaire consisted of, first, instruments for socio-demographics: gender, age, type of disorder, education level, marital status, socioeconomic level, history of familial psychiatric disorders, duration of illness, duration of hospitalization and medication. In addition, six measures were either self- or interviewer-administered to all participants. Assisted completion was adopted for patients who were unable to read the instructions, or for those who preferred this method over self-completion. differences of reporting methods across participants has proven to.

not cause bias in patient-reported outcome results [70].State and trait depression were measured using the Maryland Trait and State Depression (MTSD) scale. This scale comprises 36 items divided into 18 questions for state depression; which measures current depressive symptoms experienced during the last seven days leading to the day of the interview (e.g., “I feel sad”), and 18 questions for trait depression; which measures the recurrence of depressive symptoms through adult life for the exception of the last seven days before the interview day (e.g., “I often feel sad most of my life”), in schizophrenic patients. The criteria for major depression in the DSM IV and several instrument for measuring depression were relied upon to develop the questionnaire. Symptom severity is reported based of the frequency of its occurrence, on a scale from 0 to 4 [71]. The Cronbach alpha value for the MTSD-State depression was 0.930 while for the MTSD-trait depression was 0.947.

The Arabic version [72] of the Self-evaluation of Negative Symptoms scale (SNS) [54] was used to assess negative symptoms in the study population. The scale consists of 20 questions divided into 5 categories: social withdrawal, emotional withdrawal, alogia, avolition and anhedonia. The responses were given based on a scale: 0 (strongly disagree), 1 (somewhat agree), 2 (strongly agree). The total score can range from 0 to 40 (severe negative symptoms). The Cronbach alpha value was 0.910.

Loneliness was assessed using Wilson’s modified version of Jong-Gierveld Loneliness Scale [73]. Validated in Arabic [74], it consists of 5 closed questions: “I experience a general sense of emptiness”, “I miss having people around”, “I feel like I don’t have enough friends”, “I often feel abandoned,” and “I miss having a really good friend.” Total scores range from 0 to 5, with higher values meaning higher feeling of loneliness. The Cronbach alpha value was 0.624.

Insight was then measured using Birchwood Insight Scale (BIS) [75]. It consists of 8 questions, 2 assessing symptom awareness, 2 assessing illness awareness and 4 assessing the awareness of needing treatment. A total score 9–12 indicates a good insight, minimal scores indicate lack or bad insight. The BIS has shown to be reliable and valid for assessing insight in schizophrenia [76]. The Arabic translated version of the BIS has previously been used in Lebanese patients with schizophrenia [66]. The Cronbach alpha value was 0.755.

Internalized stigma was measured using the Internalized Stigma for Mental Illness (ISMI) scale [77] in its Arabic version [78]. It consists of 29 questions, divided into 5 subscales: alienation, stereotype endorsement, discrimination experience, social withdrawal and stigma resistance. Each item is rated on a scale: 1 (strongly disagree), 2 (disagree), 3 (agree) and 4 (strongly agree). The stigma resistance items are reverse coded and their score is obtained by subtracting the actual value from five. The highest the total score, the more intense internalized stigmatization is. The Cronbach alpha value was 0.882.

Social support was measured using the Oslo Social Support Scale (OSSS-3) [79]. It is comprised of 3 items: “How many people are so close to you that you can count on them if you have great personal problems?” with responses based on a scale from 1 to 4, “How much interest and concern do people show in what you do?” and “How easy is it to get practical help from neighbors if you should need it?” with responses’ scale from 1 to 5. The total score ranges from 3 to 14; a score between 3 and 8 indicating poor social support, between 9 and 11 moderate social support, and between 12 and 14 strong social support. The Cronbach alpha value was 0.724.

Forward and backward translation procedure: All scales were used in their Arabic translated and validated versions, except for the MTSD and the OSSS-3. These two scales have been translated in Arabic for use in the present study. The translation process was performed following rigorous recommended international guidelines [80]. Two independent psychologists who are experts in the translation field translated the two scales into Arabic. Prior to beginning their translations, all translators were made aware of the goals of the study. The lead researcher next compared the original English questionnaire to the back-translated English questionnaire with the aim of discovering differences and resolving discrepancies between both versions. Problematic questions were discussed and shared with the concerned translators for possible updates. Repeated forward-back translations were carried out until there were no longer any ambiguities [81–87].

When conducting studies, controlling the impact of multiple antipsychotics can be complicating, given that they present different formulations, potentials, dosages. A reference medication is used and the equivalent doses of antipsychotics is calculated to compare the effectiveness of the drugs and their doses. Chlorpromazine is often used as this reference medication. Chlorpromazine equivalent dose is determined as the dose of a drug that is identical to 100 mg of chlorpromazine. The minimum effective dose is the drug’s dose that is equivalent to 200–300 mg of chlorpromazine, and a high dose of drugs is equivalent to more than 1000 mg of chlorpromazine [88].

### Data analysis

Data collected was entered and analyzed using the software Statistical Package for the Social Sciences (IBM SPSS statistics v.25). Reliability using Cronbach’s alpha values for different scales were also included. As the current study has an exploratory design, we first conducted a hierarchical cluster analysis based on the Z scores for the five negative symptoms scores on the whole sample, using the Ward’s method with Euclidean distance. Ward’s method was suggested to be more appropriate for various types of data structures compared to other hierarchical algorithms [89], and the Euclidean distance, a commonly used distance measure, is known to be more suitable for numerical variables [90, 91]. The optimal number of clusters has been identified based on information from both agglomeration schedule and dendrogram. After the number of clusters have been identified, K-means clustering was used to assign each individual to the identified clusters [92]. The data was normally distributed as demonstrated by the visual inspection of the histogram, and the skewness and kurtosis varied between − 1 and + 1 [93]. Cluster group differences were tested using one-way ANOVA for continuous variables and Chi-square test for categorical variables. The ANCOVA test was used to compare three means after adjustment over other variables; adjustment was done over all variables that showed a *p* < 0.25 in the bivariate analysis. Bonferroni post hoc tests were conducted to the groups two by two. Partial eta squared (ɳ_p_^2^; representing the effect size) was also recorded. ɳ_p_^2^ values of 0.01, 0.06 and 0.14 indicate small, medium and large effects respectively [94]. *P* < 0.05 was considered statistically significant.

## Results

### Sample description

Of the 202 participants, 126 (62.4%) were males. The mean age of the patients was 56.71 ± 11.61 years (82.2% diagnosed with schizophrenia). Table 1 shows the sociodemographic and other characteristics of the patients. The mean chlorpromazine equivalent dose was 1026.54 ± 992.07.

**Table 1:** Sociodemographic and other characteristics of the participants (N = 202)

Variable	N (%)
**Gender**	
Male	126 (62.4%)
Female	76 (37.6%)
**Marital status**	
Single/divorced/widowed	185 (91.6%)
Married	17 (8.4%)
**Education level**	
Illiterate	17 (8.4%)
Primary	43 (21.3%)
Complementary	67 (33.2%)
Secondary	54 (26.7%)
University	21 (10.4%)
**Diagnosis**	
Schizophrenia	166 (82.2%)
Schizoaffective disorder	36 (17.8%)
	**Mean ± SD**
**Age (in years)**	56.71 ± 11.61
**Duration of illness (in years)**	31.56 ± 12.29
**Duration of hospitalization (in years)**	28.55 ± 12.63
**Chlorpromazine equivalent dose for antipsychotics (in mg)**	1026.54 ± 992.07

### Clusters

Data revealed a first group [n = 52 (25.7%)] characterized by moderate mean NS subscales scores; this group was labeled “moderate negative symptoms” (MNS). The second group [n = 86 (42.6%)] had the lowest mean NS subscales scores; this cluster was called “low negative symptoms” (LNS). A third group [n = 64 (31.7%)] was characterized by the highest mean NS subscales scores; thus, this group was labelled “high negative symptoms” (HNS) (Table 2).

**Table 2:** Classification of patients by clusters

	Moderate NS cluster n = 52 (25.7%)	Low NS cluster n = 86 (42.6%)	High NS cluster n = 64 (31.7%)
Social withdrawal	3.54	0.60	3.91
Emotional withdrawal	3.92	2.27	4.55
Alogia	3.37	0.85	5.00
Avolition	1.63	1.22	6.16
Anhedonia	2.73	1.33	6.22

### External validity of the three-cluster model for negative symptoms

The results of the comparison of clusters in terms of sociodemographic and other characteristics are summarized in Tables 3 and 4. Higher mean state depression, trait depression, and loneliness scores were significantly found in patients belonging to the cluster “high negative symptoms”, whereas a higher mean social support score was found in patients belonging to the cluster “low negative symptoms”.

**Table 3:** Comparison of categorical variables in terms of clusters

	Moderate NS cluster n = 52 (25.7%)	Low NS cluster n = 86 (42.6%)	High NS cluster n = 64 (31.7%)	X^2^	*p*
**Gender**				3.78	0.151
Males	31 (24.6%)	60 (47.6%)	35 (27.8%)		
Females	21 (27.6%)	26 (34.2%)	29 (38.2%)		
**Marital status**				3.76	0.204
Single/divorced/widowed	49 (26.5%)	75 (40.5%)	61 (33.0%)		
Married	3 (17.6%)	11 (64.7%)	3 (17.6%)		
**Education level**				4.48	0.804
Illiterate	5 (29.4%)	6 (35.3%)	6 (35.3%)		
Primary	11 (25.6%)	23 (53.5%)	9 (20.9%)		
Complementary	17 (25.4%)	27 (40.3%)	23 (34.3%)		
Secondary	14 (25.9%)	20 (37.0%)	20 (37.0%)		
University	5 (23.8%)	10 (47.6%)	6 (28.6%)		
**Diagnosis**				0.25	0.883
Schizophrenia	42 (25.3%)	72 (43.4%)	52 (31.3%)		
Schizoaffective disorder	10 (27.8%)	14 (38.9%)	12 (33.3%)		

**Table 4:** Comparison of continuous variables in terms of clusters

	Moderate NS cluster n = 52 (25.7%)	Low NS cluster n = 86 (42.6%)	High NS cluster n = 64 (31.7%)	X^2^	*p*	post-hoc comparisons	
Age	56.52 ± 11.09	55.65 ± 12.80	58.30 ± 10.28	0.96	0.384	-	
Duration of illness	31.52 ± 12.51	30.22 ± 11.61	33.41 ± 12.96	1.23	0.293	-	
Duration of hospitalization	27.81 ± 12.51	27.22 ± 12.86	30.97 ± 12.28	1.75	0.176	-	
Chlorpromazine equivalent dose	926.42 ± 950.75	943.73 ± 939.12	1219.17 ± 1078.48	1.79	0.171	-	
State depression	17.77 ± 13.90	17.59 ± 12.86	30.09 ± 13.24	19.25	**< 0.001**	C3 > C1; C3 > C2	
Trait depression	23.90 ± 15.10	20.59 ± 12.68	33.55 ± 12.75	17.77	**< 0.001**	C3 > C1; C3 > C2	
Loneliness	2.15 ± 1.43	1.90 ± 1.20	3.36 ± 1.34	24.56	**< 0.001**	C3 > C1; C3 > C2	
Stigma	2.27 ± 0.31	2.18 ± 0.18	2.24 ± 0.38	1.88	0.156	-	
Social support	9.29 ± 2.46	10.10 ± 2.42	7.47 ± 2.42	21.91	**< 0.001**	C3 < C1; C3 < C2	
Insight	4.52 ± 3.55	3.98 ± 3.06	4.13 ± 3.27	0.45	0.641	-	

After adjustment over all variables, the results showed a higher mean state depression, trait depression, and loneliness scores in patients belonging to the HNS cluster; the Bonferroni post-hoc analysis showed a significantly higher mean of those scores in patients with high NS compared to patients belonging to LNS and MNS clusters, respectively. Finally, a higher mean social support score in patients belonging to the cluster LNS; the post-hoc analysis revealed a significantly lower mean social support score in patients of the HNS cluster compared to patients belonging to LNS and MNS clusters, respectively (Table 5).

**Table 5:** Typology of individuals based on negative symptoms

	Moderate NS cluster n = 52 (25.7%)	Low NS cluster n = 86 (42.6%)	High NS cluster n = 64 (31.7%)	F	*p*	ɳ_p_2	Significant post-hoc comparisons
State depression	23.86	23.69	35.73	19.16	**< 0.001**	0.171	C3 > C1; C3 > C2
Trait depression	29.36	26.62	38.76	16.95	**< 0.001**	0.154	C3 > C1; C3 > C2
Stigma	2.36	2.18	2.22	1.27	3.71	0.027	C1 > C2
Insight	5.32	4.90	4.75	0.454	0.636	0.005	-
Loneliness	2.28	2.03	3.46	21.41	**< 0.001**	0.187	C3 > C1; C3 > C2
Social support	8.77	9.63	6.96	20.85	**< 0.001**	0.183	C3 < C1; C3 < C2

Results are adjusted over age, gender, education duration of illness, duration of hospitalization, total chlorpromazine equivalent dose, and type of schizophrenia.

## Discussion

Dissecting the heterogeneity of schizophrenia may help foster progress in understanding its etiology and lay the groundwork for the development of new treatment options for primary or enduring NS. In this regard, the present study adopted a cluster analysis approach to classify patients with schizophrenia and schizoaffective disorder based on the five NS domains social withdrawal, emotional withdrawal, alogia, avolition and anhedonia. A three-cluster solution was obtained based on unique NS profiles, and divided patients into low NS (LNS; 42.6%), moderate NS (MNS; 25.7%) and high NS (HNS; 31.7%). We next examined associations of the three unveiled NS clusters to clinically relevant external validity variables. Results indicated that the identified subgroups significantly differed on state depression, trait depression, loneliness and social support.

Our sample of 202 chronic inpatients was divided into three distinct homogeneous profiles (LNS, MNS, HNS) based on the five NS domains (social withdrawal, emotional withdrawal, alogia, avolition, anhedonia) used for clustering. These findings expand and corroborate previous research using various cluster analytic approaches and carried out mainly in Western countries [42]. While extensive research efforts have been devoted to dissecting the heterogeneity of schizophrenia using statistical subgrouping methods [27, 95–97], only few empirical evidence sought to determine meaningful boundaries within the disease based on NS (e.g [43–45].,). Using a similar procedure to ours, Paul et al. [52] were the first to deconstruct heterogeneity in schizophrenia through the five NIMH NS domains (i.e., anhedonia, asociality, avolition, blunted affect, and alogia) in a sample of 220 outpatients meeting criteria for schizophrenia or schizoaffective disorder from the USA. Authors indicated a four-cluster solution as optimal, with patients being divided into two subgroups with either low or severe NS levels, and two other subgroups with moderate NS levels and either increased blunted affect or diminished avolition [52]. Differences in identification of symptom clusters between this study and ours is likely, in part, due to cultural factors as well as symptom-reporting method (clinical interview vs. self-report). The largest number of our participants belonged to the LNS cluster (42.6%), which is consistent with recent previous findings by Paul et al. [52], and further endorses observations reported in prior cluster analysis studies that there exists a cluster with low or transient NS [22, 43–45].

The cluster analysis approach assumes that the three clusters of patients identified display more between-cluster that within-cluster variation [98]. We thus had as a second objective to investigate whether our three groups differed meaningfully from one another in their associations with external validators. To this end, we compared their performance on demographic (age, gender, marital status, educational level) and clinical (diagnosis, duration of illness, duration of hospitalization, antipsychotics dose) factors, as well as other assessments of state depression, trait depression, stigma, insight, loneliness and social support. Post-hoc comparisons showed that depression (state and trait), loneliness and social support could accurately distinguish the schizophrenia subgroups. Additionally, individuals in the HNS cluster had longer duration of illness, longer duration of hospitalization, and were given higher dosages of antipsychotic medication compared to those in the other clusters, but these differences did not achieve the statistical significance. Consistent with our results, a previous study using latent class growth analysis to model changes in NS over a 12-month follow-up period found that depression predicted initially high NS in a large sample of patients with first episode psychosis [99]. There is a phenomenological overlap between negative and depressive symptomatology, and a substantial lack of clarity on how to reliably assess them and how to validly distinguish between them [100]. Depressive symptoms are (more often) defined by self-report criteria [101]. Older clinician-rated measures (e.g., the Scale for the Assessment of Negative Symptoms [102]) conceptualize NS as a single construct including multiple symptoms, and do not discriminate experiential NS that are commonly seen in depression (i.e. low motivation, anhedonia and withdrawal) from expressive symptoms (i.e. blunted affect and alogia) [101]. Newer measures, such as the SNS used in the present study to assess NS, have demonstrated good divergent validity from depressive measures [54, 58, 59]. In sum, these observations are further in agreement with prior findings that, despite the theoretical overlap in clinical presentation between NS and depression (e.g., social withdrawal, anhedonia, apathy, diminished emotional expression) often leading to a diagnostic dilemma between the two entities [103, 104], they are distinct and separate symptom domains according to factor analysis studies [105]. Furthermore, a variety of depressive symptoms may overlap with certain other features common to schizophrenia, including neuroleptic induced side effects [106] or the negative effects of long-term hospitalization. Therefore, our findings should be considered tentative until confirmed by future studies involving outpatient populations and using clinical interview to assess both depression and NS.

The predicting effect of social support and loneliness is also consistent with prior research on NS, which suggests that the deficit schizophrenia group (exhibiting high NS levels) appear to suffer from a marked lack of interpersonal relatedness [107], as well as great difficulties in social contact and interest [108]. In accordance with our findings, a previous study showed that NS groups were not distinguishable by perceived level of internalized stigma [108]. Nevertheless, it was somewhat unexpected to find no differences between subgroups regarding some external validators, including level of education, duration of illness, duration of hospitalization, antipsychotics dose, as previous studies have established such associations [22, 52]. Therefore, additional future studies are necessary to elucidate the separation between clusters on these factors.

### Clinical implications and research perspectives

Our cluster analysis proposes that subtypes of schizophrenia may exist with severity-based differences in underlying NS. The HNS subgroup had the greatest levels of trait/state depression and loneliness, and the lowest levels of perceived social support. Altogether, findings advance that schizophrenia encompasses qualitatively separate NS subgroups that differ in their psychopathological profiles. Heterogeneity in schizophrenia may echo a combination of homogeneous, non-arbitrary subgroups that, when taken into account, shed light on different etiological processes and guide efforts to develop more effective and more specific treatments based on group-level characteristics [42]. Indeed, the identification of schizophrenia subgroups could assist in advancing evidence-based personalized medicine in the field of schizophrenia and related psychoses by selecting and applying treatment options appropriate for subtypes of patients with similar and unique features. Finally, the identification of depression, loneliness and social support as clinically relevant predictors offers promising avenues to develop clinical risk (and machine learning) prediction models [42].

### Study limitations

Despite its significant contribution to the international literature, this study has certain limitations that need to be acknowledged. First, our study included long-stay chronic inpatients, predominantly males (62.4%), with a long mean duration of illness (over 30 years) from Lebanon, which may affect the generalizability of our findings to outpatients, younger, female patients in early phases of illness, as well as those in other countries and cultures. Second, data was gathered at a single point in time, which prevented investigation of the stability of negative symptom profiles across phases of illness. Future longitudinal studies are required to address this point. Third, although self-evaluation of NS by the patients themselves has its advantages, it remains subjective and should be complemented by semi-structured interviews. Fourth, our external validation data did not include other important clinical variables (e.g., positive symptoms), neurocognitive symptoms, psychosocial functioning, quality of life, genetic and structural neuroimaging factors; resulting in limited information about qualitative differences across the three subgroups. More research should consider including additional external validators. Another important limitation to the present study is that it did not examine whether the links found are equivalent across all different forms of NS, such as primary vs. secondary NS, especially since these subgroups vary on some of the external validator variables including positive psychotic symptoms, substance use, social deprivation, and depression [37, 109, 110]. Although previous studies investigating NS subgroups did neither differentiate primary from secondary symptoms, nor did they control for the major sources of secondary NS (e.g [50, 52].,), we recommend that future research differentiate between these different forms of NS. In addition, our sample consists of long-stay hospitalized patients with an average duration of hospitalization of 28 years, which might have affected our findings, as residence in an institution is likely to contribute to secondary NS [111]. Prolonged hospital stay could also significantly affect perceived social support and loneliness experiences [112]. Therefore, further studies need to replicate our findings in outpatients.

## Conclusion

As far as we are aware of, this is the second study that attempts to separate clusters across the recently conceptualized five latent constructs of NS, and the first in its nature in an Arab clinical population and a developing country from the MENA region. Findings provide additional support to the categorical model of schizophrenia by confirming the existence of three alternate subtypes based on NS. The clusters identified are characterized by low, moderate or high NS, and convey meaningful information about psychopathology profiles. The determination of distinct NS subgroups within the broad heterogeneous population of people diagnosed with schizophrenia may imply that each subgroup possibly has unique underlying mechanisms and necessitates different treatment approaches. Future research is still needed to confirm or infirm these assumptions.

## Data Availability

The datasets generated and/or analysed during the current study are not publicly available due to restrictions from the ethics committee but are available from the corresponding author upon a reasonable request.
